# The complete chloroplast genome of *Phyllostachys edulis* f. *tubiformis* (Bambusoideae): a highly appreciated type of ornamental bamboo in China

**DOI:** 10.1080/23802359.2021.2018945

**Published:** 2022-01-10

**Authors:** Xinmiao Liu, Lei Liu, Lubin Li, Jinjun Yue

**Affiliations:** a Research Institute of Subtropical Forestry, Chinese Academy of Forestry, Fuyang, China; b State Key Laboratory of Forest Genetics and Tree Breeding, Key Laboratory of Tree Breeding and Cultivation of the State Forestry Administration, Research Institute of Forestry, Chinese Academy of Forestry, Beijing, China

**Keywords:** *Phyllostachys edulis* f. *tubiformis*, chloroplast genome, phylogeny, moso bamboo

## Abstract

*Phyllostachys edulis* (Carr.) H. de Lehaie f. *tubiformis* (S.Y.Wang) Ohrnberger (shengyin bamboo in Chinese) is a dwarf form of moso bamboo, which has important ornamental value. In this study, the complete chloroplast genome of *P. edulis* f. *tubiformis* was reported. The complete chloroplast genome of *P. edulis* f. *tubiformis* is a double-circular DNA of 139,678 bp in length with 38.89% G + C content, and contains 126 genes, including 84 protein-coding genes, eight ribosomal RNA (rRNA) genes, and 34 transfer RNA (tRNA) genes. Phylogenetic analysis results strongly supported that *P. edulis* f. *tubiformis* was clustered with the other infra-species of *P. edulis*, although its morphology is quite different from moso bamboo.

*Phyllostachys edulis* (Carr.) H. de Lehaie f. *tubiformis* (S.Y.Wang) Ohrnberger (shengyin bamboo in Chinese) is a dwarf form of moso bamboo (Wang 1984; Ma et al. 2014). Compared with the normal form of moso bamboo (*P. edulis*), shengyin bamboo has swollen short internodes on the middle-lower part of the stems, and the swelling and shortened internodes resemble a cascade of gold ingots (Tang and Chen 2009; Zhang et al. **2020**). Due to its unique culm shape, shengyin bamboo has important ornamental value and may play an important role in understanding the molecular mechanism of shortened internodes in *P. edulis* (Wang et al. 2018; Yue et al. 2018). In order to illustrate the phylogeny of *Phyllostachys*, we reported the complete chloroplast genome sequences of *P. edulis* f. *tubiformis* in this study.

The young and fresh leaves of *P. edulis* f. *tubiformis* were harvested in April, 2019 at the breeding base of Shengyin bamboo resources in the Yiyang (28°30′ N, 112°30’E, 58.3 m above sea level) and approved by Yiyang Research Institute of Forestry Science of Hunan Province, China. The voucher specimens have been deposited in the Research Institute of Subtropical Forestry, Chinese Academy of Forestry (http://risfcaf.caf.ac.cn/, contact: Jinjun Yue; email: yuejinjun@163.com) under the voucher number RISF-CAF-2020778. Total genome DNA was extracted with the Qiagen plant genomic DNA prep kit (Sangon Biotech, Shanghai, China), which were sequenced on the Illumina HiSeq 2500 platform in the Beijing Biomarker Technologies Corporation. Approximately 42.3Gbp raw data (nearly 20× coverage) of paired-end reads were generated with 150 bp in length. The clean reads were assembled by using GetOrganelle v1.5 (Jin et al. 2018). The seed sequence was the chloroplast sequences carried in the software seed bank, and the organelle type was embplant_Pt; other parameters were the default values. Genome annotation was performed by the GeSeq and support annotation by Chloё (Tillich et al. 2017). Inverted Repeat (IB) and trans-spliced *rps12* were also annotated. A chloroplast genome map of the annotated *P. edulis* f. *tubiformis* was drawn by OGdraw online (Lohse et al. 2013). Assembly data have been deposited in GenBank with accession numbers MW874473.

The complete chloroplast genome of *P. edulis* f. *tubiformis* is a double-circular DNA of 139,678 bp in length, including a pair of inverted repeat (IRA and IRB) regions of 21,798 bp, a large-single copy (LSC) region of 83,212 bp and a small single copy (SSC) region of 12,870 bp. The G + C content of the total genome was 38.89% (A: 30.97%, T: 30.14%, G: 19.25%, C: 19.64%), and the corresponding value of LSC, SSC, and IR regions is 36.97%, 33.17% and 44.22%, respectively. The complete chloroplast genome contains 126 genes, including 84 protein-coding genes, eight ribosomal RNA (rRNA) genes, and 34 transfer RNA (tRNA) genes. The LSC region contains 17 transfer RNA (tRNA) genes and 60 protein-coding genes, and the SSC region contains one transfer RNA (tRNA) genes and 10 protein-coding genes. About 19 genes are duplicated in the IR regions, including all four rRNA genes, eight tRNA genes, and seven protein-coding genes. Eight genes (*rpl16*, *petD*, *petB*, *atpF*, *rps16*, *ndhA*, *ndhB*, *rpl2*) contain one intron, while *ycf3* and *rps12* contain two introns.

To determine the phylogenetic status of *P. edulis* f. *tubiformis*, about 20 complete chloroplast genomes of the genus *Phyllostachys* and its related genus, which were obtained from NCBI, were used to construct phylogenetic trees. Multiple sequence alignments were performed by using MAFFT (v7.470) (Katoh and Standley 2013). The phylogenetic tree based on complete sequences was constructed by using the maximum likelihood (ML) method embedded in IQ-tree (v1.6.8) (Nguyen et al. 2015) (Figure 1). The model K3Pu + F+G4 and HKY + F+G4 were identified as the best-fit model for IQ-tree and MrBayes after the selection of ModelFinder (Kalyaanamoorthy et al. 2017).

**Figure 1.**
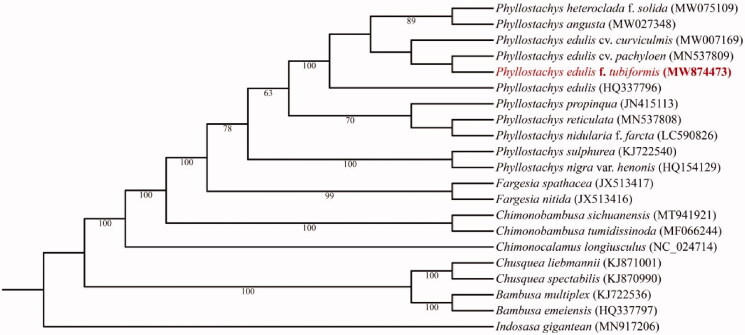
Maximum Likelihood tree of 21 species with complete chloroplast genomes. The sequence of *Indosasa gigantean* (MN917206) was used as an outgroup. Bootstrap percentages (based on 1000 replications) are shown at branching points.

Phylogenetic analysis results strongly supported that *P. edulis* f. *tubiformis* was closely related to *Phyllostachys* reticulate, clustered with the other infra-species of *P. edulis*, although its morphology is quite different from moso bamboo.

This is the first report of the complete chloroplast genome of *P. edulis* f. *tubiformis*. The chloroplast genome of *P. edulis* f. *tubiformis* will provide important genetic information for evolution, phylogeny, and taxonomy of genus *Phyllostachys*.

## Authors’ contributions

Xinmiao Liu and Lei Liu led the main bioinformatics and statistical analyses of the data. Xinmiao Liu wrote the original draft manuscript. Lubin Li and Jinjun Yue conceived of project. Jinjun Yue designed research and revised the manuscript. All authors have read and agreed to the published version of the manuscript.

## Data Availability

This genome sequence has been deposited at NCBI (http://www.ncbi.nlm.nih.gov/) with the accession No. MW874473. The associated BioProject, BioSample and SRA (Sequence Read Archive) numbers are PRJNA757557, SAMN20967582 and SRR15621974, respectively.
